# The Evolutionary unZIPping of a Dimerization Motif—A Comparison of ZIP and PrP Architectures

**DOI:** 10.3390/pathogens7010004

**Published:** 2017-12-29

**Authors:** Jian Hu, Holger Wille, Gerold Schmitt-Ulms

**Affiliations:** 1 Department of Chemistry, Michigan State University, East Lansing, MI 48824, USA; hujian1@msu.edu; 2 Department of Biochemistry and Molecular Biology, Michigan State University, East Lansing, MI 48824, USA; 3 Department of Biochemistry, University of Alberta, Edmonton, AB T6G 2M8, Canada; wille@ualberta.ca; 4 Centre for Prions and Protein Folding Diseases, University of Alberta, Edmonton, AB T6G 2M8, Canada; 5 Tanz Centre for Research in Neurodegenerative Diseases, University of Toronto, Toronto, ON M5T 2S8, Canada; 6 Department of Laboratory Medicine and Pathobiology, University of Toronto, Toronto, ON M5S 1A8, Canada

**Keywords:** ZIP metal ion transporter, prion protein, dimerization, evolution

## Abstract

The cellular prion protein, notorious for its causative role in a range of fatal neurodegenerative diseases, evolved from a Zrt-/Irt-like Protein (ZIP) zinc transporter approximately 500 million years ago. Whilst atomic structures for recombinant prion protein (PrP) from various species have been available for some time, and are believed to stand for the structure of PrP^C^, the first structure of a ZIP zinc transporter ectodomain was reported only recently. Here, we compare this ectodomain structure to structures of recombinant PrP. A shared feature of both is a membrane-adjacent helix-turn-helix fold that is coded by a separate exon in the respective ZIP transporters and is stabilized by a disulfide bridge. A ‘CPALL’ amino acid motif within this cysteine-flanked core domain appears to be critical for dimerization and has undergone stepwise regression in fish and mammalian prion proteins. These insights are intriguing in the context of repeated observations of PrP dimers. Other structural elements of ZIP transporters and PrP are discussed with a view to distilling shared versus divergent biological functions.

## 1. Introduction

Prion proteins are notorious for their central role in fatal neurodegenerative diseases in a subset of mammalian species, including humans [1,2,3]. In prion diseases, the cellular prion protein (PrP^C^) undergoes structural rearrangements to a β-sheet-rich conformer termed PrP^Sc^ (named after Scrapie in sheep, the first known prion disease). That essential role of PrP in these disorders was demonstrated by showing that the knockout of the prion protein gene (*Prnp*) renders mice refractory to acquiring the disease [4]. A side-product of mouse *Prnp* knockout studies undertaken concomitantly in several laboratories was the identification of a paralog of the prion gene, termed Doppel (*Dpl*). *Dpl* maps to a genomic region *C*-terminal to *Prnp* and, consequently, was determined to have arisen from a gene duplication event [5,6]. Further genomic sequence analyses revealed that the Shadoo (Sho) gene coded for an additional prion protein paralog [7,8]. The ancestry of this small gene family was enigmatic until 2009 when PrP was initially observed to interact with a subset of zinc transporters of the Zrt-/Irt-like Protein (ZIP) family [9], and subsequent bioinformatic analyses revealed PrP and ZIP transporters to meet several criteria that establish common ancestry [10]. The evolutionary relationship was particularly apparent in comparisons of PrP and ZIP ectodomain sequences in fish genomes, which exhibit a degree of sequence similarity and identity previously reported in pair-wise sequence comparisons of PrP and Dpl, or PrP and Sho. In contrast to ZIPs, which are multi-spanning transmembrane proteins, the prion protein is anchored in the membrane by a glycosylphosphatidylinositol (GPI) anchor, a shift in topology also observed in other protein families [11,12]. Consistent with the view that the prion protein founder gene represented a truncated ZIP gene, such a shift in topology can be experimentally induced when a gene coding for a transmembrane protein is truncated at the 3′ end of its first transmembrane domain [13]. 

**Figure 1:**
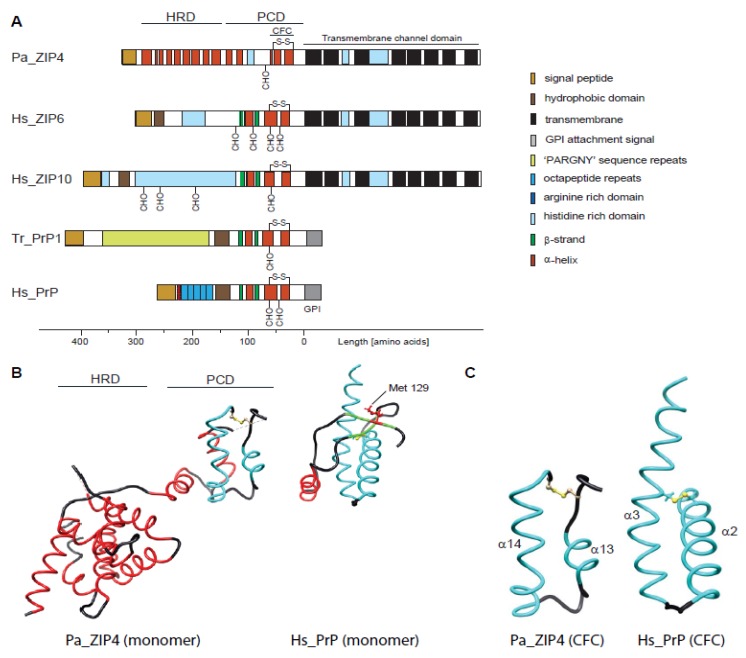
Comparison of molecular architectures of Zrt-/Irt-like Proteins (ZIP)s and prion protein (PrP) molecules, comprising either helix-rich domains (HRDs) or disordered domains at their *N*-terminus and a separate module consisting of helix-turn-helix motifs adjacent to the plasma membrane. (**A**) Domain organization of selected members of the ZIP/PrP protein family. The depiction of all proteins was centered on the predicted site of their insertion into the outer face of the plasma membrane. Pa, *Pteropus alecto*; Hs, *Homo sapiens*; Tr, *Takifugu rubripes*. (**B**) Side-by-side views of the bat (Pa) ZIP4 ectodomain monomer (PDB: 4x82; Zhang et al., 2016; Nat Commun) and the human PrP NMR structure (PDB: 1qm0; Zahn et al., 2000; PNAS). Homologous α-helices are colored in cyan. Just for reference, the rendering also depicts the position of the side-chain of methionine-129 within the first short β-strand in PrP^C^. (**C**) Comparison of CFC domains of bat ZIP4 (PDB: 4x82) and human PrP (PDB: 1qm0). As the available bat ZIP4 ectodomain structure does not contain residues *C*-terminally to the CFC, its structure ends on the respective cysteine, but the PrP structure extends to the *C*-terminus.

Whereas the biology of the prion protein in health and disease has been extensively studied and reviewed [3,14,15], considerably less is known about ZIP transporters, which are coded by members of the solute carrier 39a (Slc39a) gene family. In humans and mice, this family comprises 14 genes, whose gene products appear to be tasked with the import of zinc and other divalent cations into the cytosol, either from the extracellular space or from intracellular compartments. Autosomal recessive mutations in ZIP4 and ZIP13 genes have been linked to *Acrodermatitis enteropathica*, a rare zinc deficiency syndrome [16], and a form of Ehlers-Danlos syndrome characterized by a skeletal dysplasia that mainly affects the spine, and also causes developmental deformations of the hands [17]. The latter symptoms speak to an emerging pattern of ZIP-dependent phenotypes that point toward roles of these proteins in specific morphogenetic programs. In particular, members of the so-called LIV1 subfamily of ZIPs, featuring ectodomains with homology to PrP [10], stand out in this way (Figure 1A). For instance, ZIP6 and ZIP10, the ZIP transporters most closely related to PrP, were shown to contribute to the mammalian oocyte-to-egg transition [18]. Moreover, the morpholino-based knockdown of ZIP6 or ZIP10 caused an embryonic arrest in zebrafish that exhibited characteristics of an impaired epithelial-to-mesenchymal transition (EMT) [19,20]. Test embryos exhibited a phenotype reminiscent of a similar impairment to that observed following the knockdown of PrP in the same paradigm [21]. The striking overlap in ZIP6- and ZIP10-dependent phenotypes was recently resolved by data, which clarified that these proteins form a functional heteromer [19,22]. The ability of these ZIPs to interact directly may also account for their original appearance amongst a short list of PrP interacting proteins [9]. This theory assumes that PrP inherited structural features responsible for interactions amongst these ZIPs from a common ancestor. It is currently unclear whether direct or third-party interactions underlie shared links of PrP and ZIPs to EMT. Recent work in NMuMG cells, a mammalian EMT model, put a spotlight on the neural cell adhesion molecule (NCAM1), by showing that not just PrP [23,24,25] but also the ZIP6-ZIP10 heteromer [22] predominantly interacts with this cell adhesion molecule. However, although both PrP and ZIP6-ZIP10 affect post-translational modifications on NCAM1 during its involvement in EMT, they do so in different and perhaps complementary ways. More specifically, whereas signaling downstream of PrP was shown to control the transcription of the sialyltransferase (ST8SIA2) that mediates NCAM1 polysialylation [26], the ZIP6-ZIP10 heteromer appeared to control NCAM1 phosphorylation at a specific cluster of cytosolic phosphoacceptor sites through its recruitment of GSK3 [22]. 

What are the structural features that govern these similarities and differences between PrP and its closest ZIP family members? Until recently, high-resolution structural data were only available for recombinantly expressed PrP [27,28,29], but not for ZIPs. According to these data, prion proteins from various species are composed of a disordered *N*-terminal domain and a folded *C*-terminal domain characterized by three α-helices and a short two-stranded β-sheet. The two most *C*-terminal α-helices form a conserved helix-turn-helix fold that is stabilized by an internal disulfide bridge and can be post-translationally modified by up to two complex *N*-glycans. Attempts to solve the structure of a fish prion protein, which presumably would be more closely related to ZIP ectodomain structures, were not met with success [30]. Finally, the first high-resolution crystal structure of a ZIP ectodomain [31,32] became available in the past couple of years. This was followed by the discovery of a separate structure of a prokaryotic ZIP containing the transmembrane domain conserved in the ZIP family [32]. Although the ZIP ectodomain structure is from ZIP4, a relatively distant PrP relative [10], the sequence of its membrane-adjacent domain is sufficiently similar to ZIP6 and ZIP10 to be of interest in this context. Here we describe this structure, compare it to PrP, and discuss its significance for understanding the biology and evolution of mammalian prion proteins.

## 2. Main

### 2.1. Comparison of Molecular Architectures of ZIP and Prion Proteins

The ZIP4 ectodomain (from *Pteropus alecto*, the fruit bat) was solved at a resolution of 2.85 Å and was shown to be composed of two structurally independent subdomains. Its *N*-terminal subdomain of 156 amino acids, bearing no sequence homology to PrP, folds into a globular cluster of nine α-helices, termed helix-rich domain (HRD). The HRD is connected through a flexible linker to a folded *C*-terminal domain comprising amino acid residues 192–322. The latter featured two helix-turn-helix folds composed of helices α10–α11 and α13–α14 that were connected to each other by a disordered histidine-rich segment (residues 232–255) and a short α12 helix (Figure 1B). A striking feature of the ZIP4 ectodomain is that it was crystallized as a dimer, held together by extensive interactions among the hydrophobic residues on a large interface including the ‘PAL’ sequence motifs present in the middle of helix α14 of interacting protomers. Hence, it was suggested to refer to the *C*-terminal subdomain as the PAL-motif containing domain (PCD). When comparing the ZIP4 PCD to PrP, the most apparent shared characteristic is the *C*-terminal helix-turn-helix fold, which in PrP comprises its helices α2 and α3 (often referred to as helices B and C). However, helix α2 in PrP is longer than α13 in ZIP4, thereby spanning homologous sequence segments that encompass ZIP4 PCD helices α12 plus α13 (Figure 1C). More *N*-terminal sequence elements present in mammalian prion proteins, including helix α1 (helix A) and the short two-stranded β-sheet observed in this sequence region, cannot be aligned to structural elements within ZIP4. While these elements are well-conserved within mammalian PrP, they fall within a sequence segment that is highly divergent not only in ZIPs but also in fish prion proteins. The diversity of this region can be attributed to the presence of repetitive elements that are prone to contract and expand throughout evolution but may also indicate that this region is not essential for a shared core function of members of this protein family. In the ZIP4 PCD, a relatively short and disordered histidine-rich fragment (31 amino acids) is present between the pair of helix-turn-helix folds. The corresponding region is expanded in the subbranch of ZIPs closer related to PrP, encompassing 89 and 232 residues in human ZIP6 and ZIP10, respectively. As the respective *N*-terminal domain of mammalian PrP^C^ is known to be highly disordered, one may speculate that *N*-terminal regions of related ZIPs might also not acquire a particular fold. This may indeed be the case for ZIPs 6 and 10, whose histidine-rich fragments, preceding the second helix-turn-helix fold (α13 and α14 in ZIP4 PCD), are predicted to be unstructured, and which have no sequences that correspond to the HRD in ZIP4.

### 2.2. Disappearance of CPAL Motif in the Prion Protein Subbranch of ZIP-PrP Protein Family

**Figure 2:**
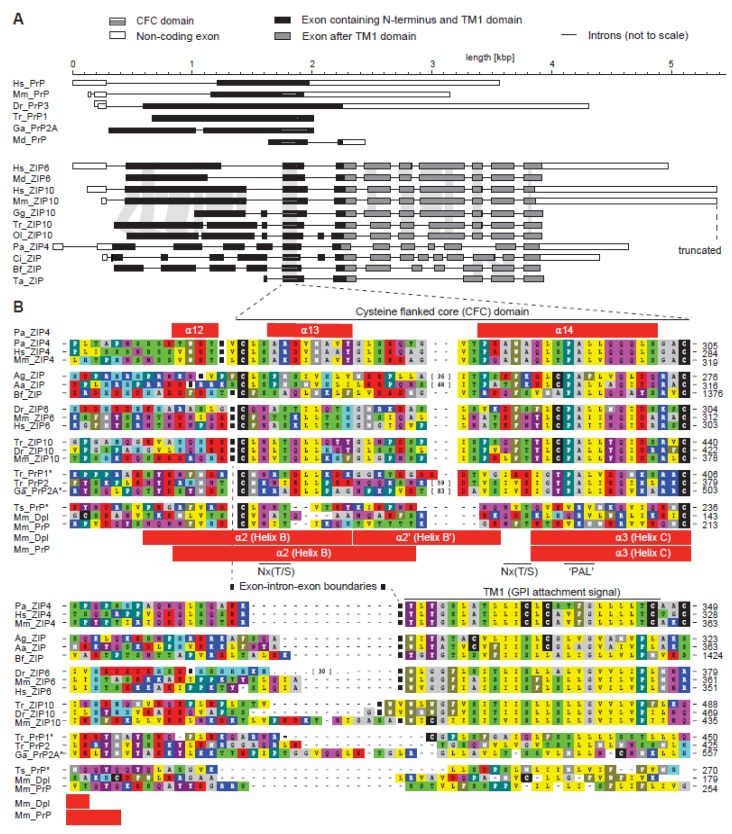
The cysteine-flanked core (CFC) domain represents an ancient intron-flanked module, from which the PAL motif gradually disappeared in the prion protein subbranch of this protein family. (**A**) Intron–exon gene architecture of selected ZIP and PrP sequences. The organization of most prion protein genes resembles the one shown for human PrP. Examples in this panel represent a wide breadth of available architectures. (**B**) Multiple sequence alignment of selected CFC sequences illustrates the gradual loss of the extended CPALL motif in fish and mammalian prion proteins. The selection of sequences was made with a view to best illustrate trends and features described in the main text. Note the absence of the cysteine preceding the PAL motif in fish PrP sequences, and the replacement of the PAL motif in mammalian PrP. Species abbreviations: Hs, *Homo sapiens*; Mm, *Mouse musculus*; Dr, *Danio rerio*; Tr, *Takifugu rubripes*; Ga, *Gasterosteus aculateus*; Md, *Mouse domestica*; Gg, *Gallus gallus*; Ol, *Oryzias latipes*; Pa, *Pteropus alecto*; Ci, *Ciona intestinalis*; Bf, *Branchiostoma florida*; Ta, *Trichoplax adhaerens*; Ag, *Anopheles gambiae*; Aa, *Anopheles aegypti*; Ts, *Trachemys scripta*. Both panels are derivatives of original Figure 2 and Figure 3 in reference [33] used under CC BY.

The ZIP4 ectodomain structure draws attention to the membrane-adjacent helix-turn-helix fold, which appears to be a core element shared between homologous members of the ZIP/PrP protein family (Figure 1C). Extensive sequence comparisons of ZIP genes across a multitude of organisms revealed this sequence segment to have evolved approximately 750 million years ago in a ZIP ancestral gene around the time when metazoans evolved [33]. The approximate timing of this event can be inferred from the presence of homologous exons in the genomes of basal metazoan organisms, including *Trichoplax adhaerens*, a marine organism with a body plan lacking organs. Whereas the exon-intron structure of ZIP genes is generally quite diverse, the genomic segment coding for this core region is invariably flanked by introns in metazoans (Figure 2A). Consequently, the absence of these flanking introns in all PrP sequences represented one of the strongest pieces of evidence in support of the conclusion that the prion protein founder gene must have evolved by retroinsertion of a spliced transcript of an ancient ZIP gene [33]. Additional elements by which this segment is recognizable include a pair of highly conserved cysteines (hence its designation as the cysteine-flanked core (CFC) domain) and the aforementioned PAL sequence. Close comparison of PrP and ZIP ortholog sequences bearing the PAL motif establish that only the proline in this motif is highly conserved, the second position is very often occupied by a small amino acid (A/G), and the third position by a hydrophobic one (mostly L/I/V). The motif is often preceded by a cysteine residue and is followed by another hydrophobic amino acid (mostly L/I/V). A regression of this extended CPALL motif appears to have occurred throughout PrP evolution, i.e., fish prion proteins lack the cysteine residue, which is present in its closest ZIP relatives, and a full replacement of the PAL motif with a sequence stretch characterized by polar amino acids occurred in mammalian PrP genes (Figure 2B). The CFC also frequently encompasses Nx(T/S) glycan acceptor sites, which, in a subset of prion proteins [34] and close ZIP relatives [35], were shown to be glycan-occupied, but nonetheless cannot be considered a core feature of the CFC domain. There is another variation to the CFC that can be observed in a subset of members of the ZIP/PrP family, namely a relatively large sequence insertion at a site predicted to form the ‘turn’ within the helix-turn-helix fold. Such insertions are found in ZIP orthologs of some insects (e.g., the mosquito *Anopheles aegypti*) and a subset of fish prion proteins (e.g., PrP2 from pufferfish, *Takifugu rubribes*). Consistent with the interpretation that the respective region in the native folds of these proteins must be able to accommodate relatively large additional structures, the second *N*-glycan acceptor site in mammalian PrP also maps to this ‘turn’. One may speculate that the lack of the PAL motif in mammalian PrP is linked to the insertion of this second *N*-glycan acceptor site. For example, it may either functionally replace the PAL motif (see below) or offer some other unknown evolutionary fitness adaptation.

### 2.3. The CPAL Motif Represents a Dimerization Interface

**Figure 3:**
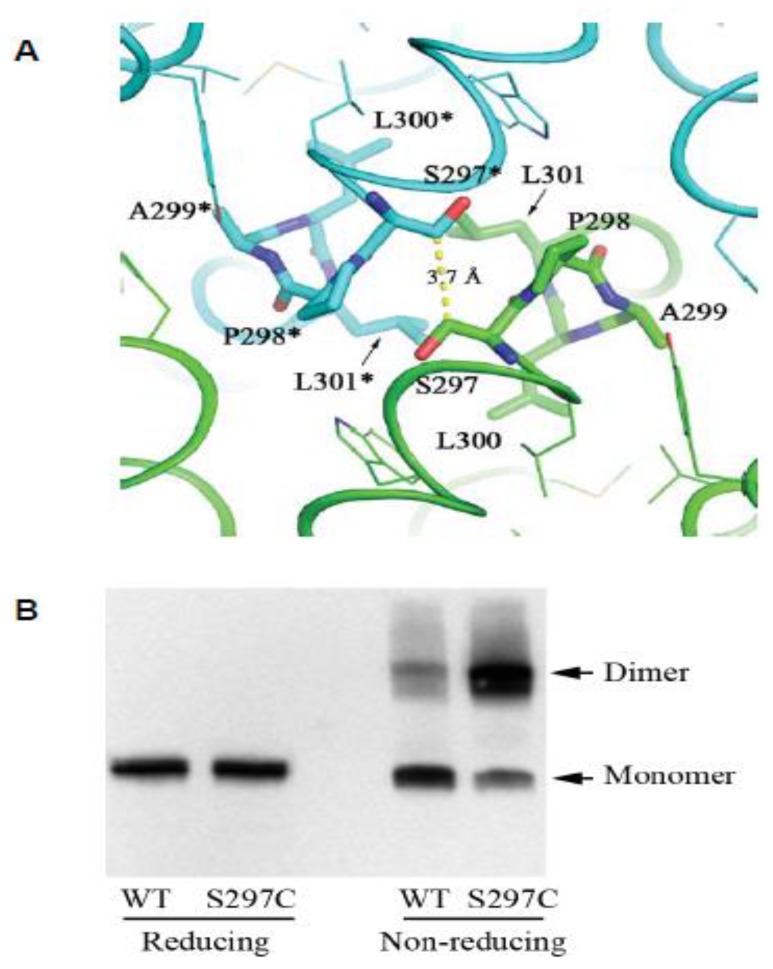
The CPAL motif present in LIV-1 and fish PrP sequences represents a dimerization interface. (**A**) Structural model of human ZIP4-ECD dimerization interface based on the crystal structure of *Pteropus alecto* ZIP4-ECD. The two protomers are colored in green and cyan, respectively. The residues in the ‘SPAL’ sequence are shown in stick mode. The ‘*’ symbol indicates the residues from the other protomer. The yellow dashed line shows the distance between the C_β_ atoms of the two interacting S297 residues. (**B**) Validation of the dimerization interface in (**A**) by substitution of S297 with a cysteine residue in the full-length human ZIP4 expressed in HEK293T cells. Both panels are derivatives of the original Figure 3C, D in reference [31] used under CC BY.

In the context reviewed here, perhaps the single most interesting insight revealed by the ZIP4 ectodomain structure is the discovery of the ZIP4 dimer interface as being centered on the hydrophobic PAL motif (Figure 3A). Several lines of evidence suggested for some time that ZIP proteins comprising a CFC domain can assemble into dimers in vitro, and may also exist as dimers in vivo. For example, recombinant ZIP5 and ZIP13 ectodomains were observed to purify as homodimers [36,37]. Additional combinatorial complexity of ZIP protein structures may exist due to the formation of heterodimers. The aforementioned ZIP6–ZIP10 heteromer is the first example of this phenomenon (note that current data are consistent with the latter complex representing a heterodimer. However, a rigorous validation of the stoichiometry of this interaction has not been made, hence its tentative designation as a heteromer [19,22]). As all of the above proteins share a homologous PAL motif, it is to be expected that this motif will also be central to their dimerization interface. When the recombinant ZIP4 construct was truncated *C*-terminal to its helix α11, the expressed protein no longer eluted as a dimer on size exclusion chromatography, indicating that the CFC is indeed essential for dimerization [31]. Moreover, a serine-to-cysteine replacement of the amino acid that precedes the ZIP4 PAL motif, thereby mimicking the predicted dimerization interface of the ZIPs that naturally harbor a cysteine in this position, caused the mutant to migrate in a Western blot as an SDS-resistant dimer (Figure 3B). This finding is intriguing, as it suggests the enhanced stability of the mutant dimer is due to the respective cysteine residues in the interacting monomers forming a disulfide bridge *in trans*. Consistent with this interpretation, the protein shifted to monomeric molecular weight, when the same samples were subjected to disulfide bridge reducing conditions prior to their SDS-PAGE separation. Additional evidence supporting this dimerization model came from the biochemical characterization of the ectodomain of ZIP14 (from *Pteropus alecto*), which naturally possesses a cysteine residue immediately preceding the conserved proline residue. Again, as for the aforementioned artificial ZIP4 mutant, a disulfide bond-mediated dimer was observed in the SDS-PAGE analysis, and a cysteine-to-serine substitution led to a monomeric species under non-reducing condition. These results corroborate the conclusion that the PAL motif-centered dimerization represents a universal model for ZIPs containing this motif in their ectodomains.

Whereas there seems to be good agreement on the predominant existence of ZIPs comprising a CFC domain as dimers, the same cannot be said for PrP, although PrP dimers were repeatedly reported. For instance, a 60 kDa PrP dimer was initially observed in murine neuroblastoma cells expressing hamster PrP [38]. Extensive subsequent recombinant work with various PrP constructs, which most often relied on in vitro refolding steps, revealed the protein to give rise to monomeric NMR structures [28]. The folding repertoire of PrP is more complex though, as the protein could be observed to crystallize as a monomer or dimer [39]. Curiously, subtle differences in the protein sequence (e.g., single amino acid mutations or the presence of the M129V polymorphism) were critical for whether the protein crystallized as a non-swapped or swapped dimer, and seemed to determine the specific architecture of the dimer interface [38,40]. Although the aforementioned studies corroborated the notion that PrP can adopt a surprising diversity of conformational states, the in vivo relevance of these states is difficult to gauge. Cell-based studies with *N*-glycosylated wild-type PrP^C^ observed a monomer–dimer equilibrium on the basis of crosslinking experiments, size exclusion chromatography, and enzyme-linked immunosorbent assay analysis [41]. Similarly, expression of cattle, hamster and human PrP in baculovirus-transduced insect cells led to dimeric PrP [42]. It is currently not understood if differences in the folding behavior of PrP in bacterial or eukaryotic cells merely reflects a need for a eukaryotic cellular protein folding environment, indicates that the attachment of *N*-glycans is critical for dimer formation, or has other causes. That dimerization might play a critical role in PrP’s biogenesis and trafficking was corroborated by fusing it to the dimerization domain of the FK506 binding protein (Fv). When dimerization was induced by the addition of an Fv dimerization ligand, the authors observed profound increases in the levels of PrP that reached the cell surface [43,44]. 

Currently available data on dimerization motifs for mammalian PrP indicate that these are non-homologous to the ZIP4 dimer interface discussed above and instead rely on structural features not shared with this ZIP transporter. It remains to be seen to which extent the structures of more closely related ZIPs will be informative for understanding this aspect of the biology of mammalian PrP. 

To date, the smallest extensively studied PrP deletion construct known to convert and transmit prion-like disease in mice was of 106 amino acids (Δ23–88, Δ141–176) [45]. This expression product comprised the entire helix CFC domain, which in human begins at amino acid 179. However, although the CFC is a critical component of disease-associated prion proteins, and appears to be retained in their proteinase-resistant core of 27 to 30 kDa (PrP27-30), the study of ZIPs may not help us elucidate their structures (the reader is reminded that even fish PrP or the mammalian paralogs Dpl and Sho have not been shown to undergo prion-like conversion). Rather, we anticipate that comparative analyses of PrP and ZIPs may continue to help elucidate the physiological function of PrP^C^, and may hold a key to understanding the molecular mechanisms by which PrP affects its next neighbors. 

Based on the striking conservation of the PAL motif in fish PrP sequences, our prediction is that the latter not only exist naturally as dimers, but also adapt the interface seen in ZIP4. Dramatic effects of point mutations on the ability of ZIP4 to fold and reach the cell surface [16,31] and a strong interaction of the ZIP6–ZIP10 complex with calreticulin (an ER resident chaperone) suggest that the assembly of ZIPs might be intricate. It then may not come as a surprise that fish PrP was observed to be refractory to recombinant protein refolding protocols that repeatedly produced high-quality NMR structures for mammalian prion proteins [30]. It is tempting to speculate that a move to a eukaryotic expression system, possibly augmented by a tyrosine-to-cysteine replacement of the tyrosine preceding the ‘PAL’ motif present in fish prion sequences, will lead to better-behaved fish PrP expression products [30].

## 3. Conclusions

Studying the physiological function of homologous segments within ZIPs may hold a key to understanding elusive aspects of the biology of PrP. Nonetheless, because ZIP4 is a relatively distant PrP relative, there are limits to the extent insights into its biology will be informative for understanding PrP. However, this overall approach should become more valuable once structures for the ZIP6–ZIP10 heteromer become available. Already, this general comparative approach has precipitated research that revealed a role of PrP in EMT [26]. It also was instrumental for understanding that the PrP–NCAM1 interaction is unlikely to have evolved following the split of PrP sequences from its ZIP relatives, but was most likely inherited by PrP and the ZIP6–ZIP10 complex from a common ancestor [22]. Currently missing are insights into the molecular workings of PrP and the molecular mechanisms by which its presence influences its interactors. We anticipate that major advances in this direction will be made once the ZIP6–ZIP10 complex can be functionally interrogated to dissect how structural elements within this complex affect its function.

## Abbreviations

Aa	*Anopheles aegypti*
Ag	*Anopheles gambiae*
AE	*Acrodermatitis enteropathica*
Bf	*Branchiostoma florida*
CDF	cation diffusion facilitator
CFC	cysteine-flanked core
Ci	*Ciona intestinalis*
CTD	carboxy-terminal domain
Dm	*Drosophila melanogaster*
Dr	*Danio rerio*
ECD	extracellular domain
Ga	*Gasterosteus aculateus*
Gg	*Gallus gallus*
GPI	glycosylphosphatidylinositol
HRD	helix-rich domain
Hs	*Homo sapiens*
LZT	LIV-1 subfamily of ZIP zinc transporters
Md	*Monodelphis domestica*
Mm	*Mus musculus*
Ol	*Oryzias latipes*
ORF	open reading frame
Pa	*Pteropus alecto*
PCD	PAL-motif containing domain
PL	prion-like
PrP^C^	cellular prion protein
SLC	solute carrier
Ta	*Trichoplax adhaerens*
TM	transmembrane
Tr	*Takifugu rubripes*
Ts	*Trachemys scripta*
